# The complete chloroplast genome of *Rhododendron platypodum* (Ericaceae): an endemic and endangered species from China

**DOI:** 10.1080/23802359.2020.1860714

**Published:** 2021-01-19

**Authors:** Li-Hui Ma, Hao-Xiang Zhu, Chao-Ying Wang, Ming-Yang Li, Hai-Yang Wang

**Affiliations:** a Key Laboratory of Horticulture Science for Southern Mountainous Regions, College of Horticulture and Landscape Architecture, Southwest University, Chongqing, China; b Chongqing City Management College, Chongqing, China

**Keywords:** Chloroplast genome, *Rhododendron platypodum*, phylogenetic analysis, Ericaceae

## Abstract

*Rhododendron platypodum* Diels (Ericaceae) is a Chinese endemic and endangered species with high ornamental value. Here the complete chloroplast (cp) genome of *R. platypodum* was assembled and characterized. The cp genome is in a total length of 201,047 bp with the typical quadripartite structure of angiosperms, containing two inverted repeats (IRs) of 44,650 bp separated by a large single-copy (LSC) region of 109,134 bp and a small single-copy (SSC) region of 2613 bp. The whole cp genome of *R. platypodum* contains 143 genes, including 93 protein-coding genes, 42 transfer RNA genes and 8 ribosomal RNA genes. Phylogenetic analysis based on the coding sequences of cp genome within the Ericaceae family suggests that *R. platypodum* is closely related to *R. delavayi*.

*Rhododendron platypodum* Diels is a Chinese endemic and endangered species with high ornamental value (Wu et al. 2005). Because of its narrow distribution (mainly in Chonging) and small population, this species has been classified as Vulnerable (VU) in Chinese Red List (http://www.mee.gov.cn) and has been listed as a key protected species in Chongqing Municipality. A good knowledge of genomic information would contribute to the protection of this species. Herein, the complete chloroplast (cp) genome sequence of *R. platypodum* was deciphered and the phylogenetic relationship with related species was detected.

Fresh and clean leaves of *R. platypodum* were collected from Wulong, Chongqing, China (N29°11′47″, E107°27′26″, 1884 m). The voucher specimen (LH Ma Wulong 2020516) was deposited in the herbarium of Southwest University (HWA). The total genomic DNA was extracted and used for sequencing on Illumina HiSeq 4000 platform at the Beijing Novogene Bioinformatics Technology Co., Ltd. (Nanjing, China). About 2 GB raw data were used to assemble the complete cp genome using SPAdes (Bankevich et al. 2012). The plastid genome of *R. delavayi* (MN413198) was used as reference for further adjustments. The complete genome sequence was annotated using PGA (Qu et al. 2019) with manual adjustments. The sequence of cp genome was deposited in GenBank (accession numbers MT985162).

The cp genome of *R. platypodum* is 2,01,047 bp in size with the typical quadripartite structure of angiosperms, containing two inverted repeats (IRs) of 44,650 bp separated by a large single-copy (LSC) region of 1,09,134 bp and a small single-copy (SSC) region of 2613 bp. The total GC content of the whole sequence is 35.9%. The cp genome of *R. platypodum* contains 143 genes, including 93 protein-coding genes (16 with two copies), 42 transfer RNA genes (six genes with two copies, one gene with three copies and one gene with five copies), and 8 ribosomal RNA genes (4 with two copies). Among the 111 unique genes, 13 had one intron (*trnI-GAU, trnA-UGC*, *ndhA*, *rps16*, *ndhB*, *petD*, *petB*, *rpl16*, *trnG-UCC*, *atpF*, *trnV-UAC*, *trnL-UAA*, *trnK-UUU*), and 2 had two introns (*rps12*, *ycf3*).

To investigate the phylogenetic position of *R. platypodum* in Ericaceae, the cp genome of another twenty species belong to Ericaceae and Clethraceae (outgroups) were obtained. The twenty protein coding genes (*accD*, *clpP*, *infA*, *matK*, *rpl14*, *rpl16*, *rpl22*, *rpl23*, *rpl32*, *rpl33*, *rpl36*, *rps2*, *rps3*, *rps4*, *rps7*, *rps8*, *rps11*, *rps14*, *rps18*, *rps19*) of cp genome of these species were aligned with MAFFT (Katoh and Standley 2013). The maximum-likelihood (ML) and Bayesian inference (BI) phylogenetic trees were reconstructed using RAxML (Stamatakis 2014) and MrBayes (Ronquist et al. 2012). The ML and BI analyses generated the same tree topology (Figure 1). The result showed that *R. platypodum* was closely related to *R. delavayi*. The complete cp sequence of *R. platypodum* reported here will provide a useful resource for further study of evolutionary and conservation genetics of this species.

**Figure 1.**
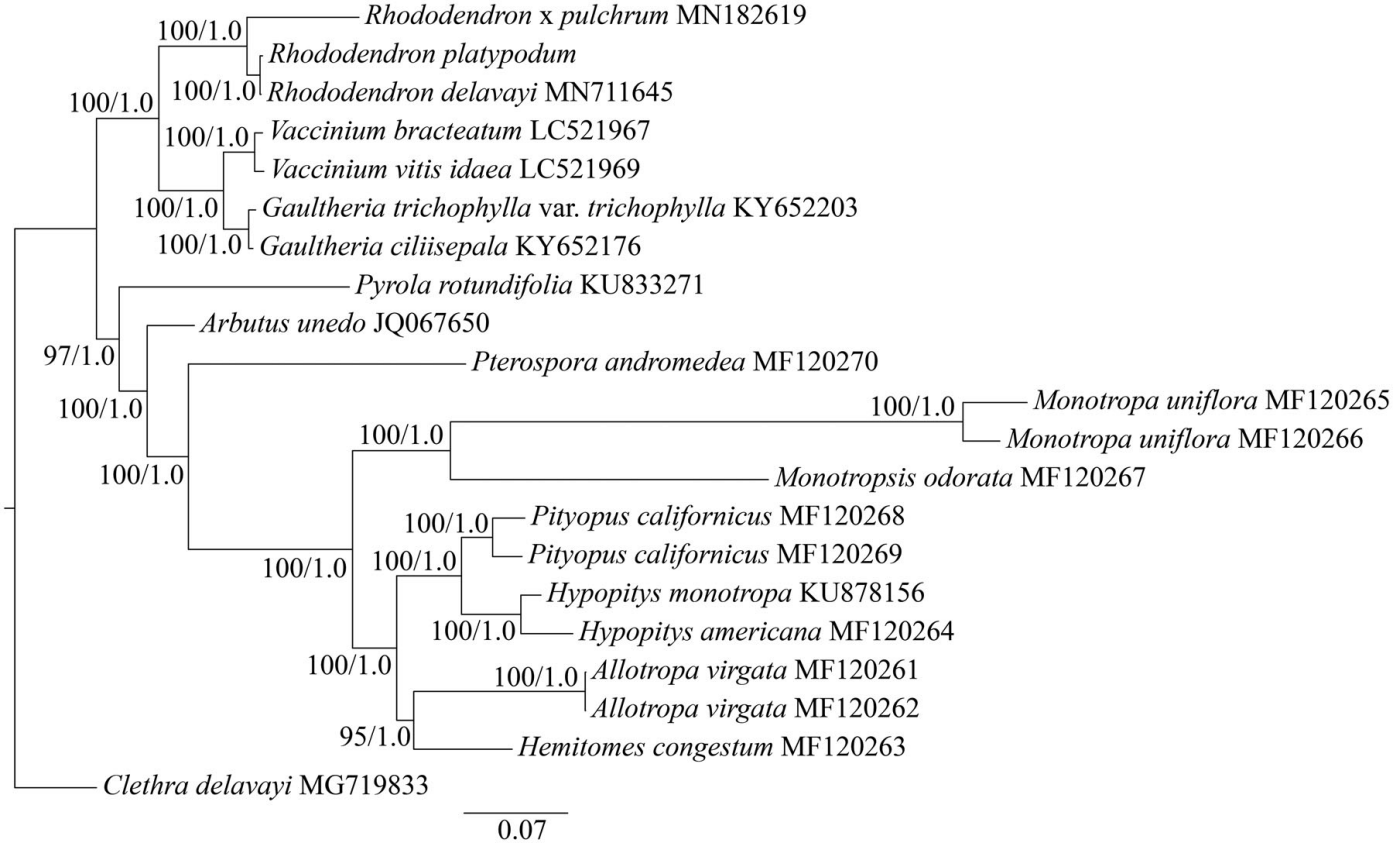
Phylogenetic tree of Ericaceae based on twenty protein coding genes of chloroplast genome. Numbers at nodes correspond to ML bootstrap percentages (1000 replicates) and Bayesian inference (BI) posterior probabilities. All of the chloroplast genome sequences are available in GenBank, with the accession numbers listed right to their scientific names.

## Data Availability

The data that support the findings of this study are openly available in GenBank of NCBI at https://www.ncbi.nlm.nih.gov, reference number MT985162.
